# Preschool or/and kindergarten? The long-term benefits of different types of early childhood education on pupils’ skills

**DOI:** 10.1371/journal.pone.0289614

**Published:** 2023-11-29

**Authors:** Yalin Tang, Renfu Luo, Yaojiang Shi, Gang Xie, Siwei Chen, Chengfang Liu

**Affiliations:** 1 China Center for Agricultural Policy, School of Advanced Agricultural Sciences, Peking University, Beijing, 100871, China; 2 Center for Experimental Economics in Education, Shaanxi Normal University, Xi’an, Shaanxi, 710119, China; Southwest University, CHINA

## Abstract

**Background:**

Developing countries have witnessed great progress in early childhood education (ECE) enrollment rate over the past three decades. Preschool and kindergarten are the two most common types of ECE in developing countries. Questions remain as to which of the two types of ECE is more effective in promoting child development in developing countries, including both cognitive and non-cognitive skills. The objective of this paper is to examine the long-term benefits of attending preschool or/and kindergarten on pupils’ cognitive and non-cognitive skills in rural China.

**Methodology:**

We pooled data from two large-scale surveys conducted by the authors themselves at 136 rural primary schools in 20 counties from three provinces in northwestern China in 2009. The final study sample consisted of 9,839 pupils who both reported their ECE experience and completed cognitive and non-cognitive tests. We measured pupils’ cognitive skills by standardized math test scores and grade retention, and their non-cognitive skills by both self-reported self-efficacy, mental health, and teacher-reported behaviors. Inverse Probability Weighting (IPW) was used to balance the pre-treatment variables between the treatment (Any ECE, Preschool Only, Kindergarten Only, or Preschool+Kindergarten) and comparison (No ECE) groups.

**Results:**

Results from IPW show that compared with their peers without any ECE experience, pupils with any ECE experience perform better in cognitive skills (0.118 standard deviations (s.d.) increase in the TIMSS, 7.1 percentage point (pp) decrease in the probability of grade retention) but not in non-cognitive skills. By ECE types, attending kindergarten only is associated with a 0.150 s.d. increase in the TIMSS, a 7.0 pp decrease in the probability of grade retention, and a 0.059 s.d. decrease in the index of behavioral problems of pupils. Moreover, attending both preschool and kindergarten predicts a lower probability of grade retention, but attending preschool only has few benefits. Heterogenous analyses suggest that the long-term benefits of ECE are more prominent among the Han pupils from households with higher socio-economic status.

**Conclusions:**

Our findings imply that increasing access to ECE can be an effective instrument to improve pupils’ skills in less-developed rural areas of China, especially their cognitive skills. Among different types of ECE, attending kindergarten contributes more to pupils’ skill development in rural China than other types. We call for strengthened efforts to ensure equal access to quality ECE for preschool-aged children in rural China.

## Introduction

Developing countries have witnessed a great progress in the expansion of early childhood education (ECE) enrollment rate over the past three decades [1]. Recognizing the important role of ECE in alleviating congenital development disadvantages of disadvantaged children and promoting human capital development throughout the whole life cycle [2,3], many developing countries, such as Argentina, Indonesia, Egypt and China, have expanded ECE programs aimed at promoting children’s both cognitive and non-cognitive skills [4–7], especially those for disadvantaged children or in underdeveloped areas [5,7]. In this context, the growth rate of ECE enrollment in many developing countries has been far ahead that in developed countries in recent years, such as that in East Asia and the Pacific (from 22% in 1991 to 62% in 2011), compared with a relatively slight increase in North America and Western Europe from 73% to 85% during the same period [1]. Among them, China has contributed a lot to the growth in ECE enrollment in the Asia-Pacific region (from 32% in 1992 to 88% in 2021) [8], which is mostly driven by the increase in rural areas [7].

The expansion of ECE in developing countries takes various forms, among which preschool and kindergarten work as two major types. Different from the United States where kindergarten works as part of the K-12 education, in many developing countries including Indonesia, Egypt, Algeria, Kenya, and China [5,6,9–11], kindergarten plays the role of providing ECE service for children aged five or six years old. Meanwhile, preschool, named as playgroup in Indonesia or “Youeryuan” in China, serves as a relatively more formal form of ECE for children no more than six years old [6,12]. Given the various ECE types, it is essential to understand whether all types of ECE play their roles similarly, or whether different types of ECE are more or less effective in promoting the skills of children, which would help inform the ECE expansion policies in developing countries.

Why the benefit of ECE might differ by its type? The ecological model proposed by Bronfenbrenner [13] can help provide some explanations to some extent. First, the school and classroom environment belong to the micro-environment where children live [13]. Specific environments, which include multiple dimensions, are found to predict specific skills of children significantly [14,15]. For instance, it is shown that structured literacy and math learning curriculums can help explain the benefits of ECE programs on children’s cognitive skills [12,16]. In addition to the curriculum dimension, teachers’ pedagogy also composites the classroom environment together, and high-quality pedagogy of teachers is shown to predict child cognitive skills significantly [17]. In the meantime, sufficient and quality teachers work as the foundation to achieve and maintain a well-organised and high-quality school or class environment [17–19]. Even given a similar school or class environment, differences in the duration children spending in the above environments might explain the differences in child development [16]. However, different types of ECE differ greatly in the above settings, which might lead to heterogenous benefits on child development. Drawing on data from the United States, Coley et al. [19] found that public centers and Head Start programs provided children with not only the most qualified teachers but also the most enriching learning activities, with private centers showing moderate quality and home ECE very low levels of quality, in the meantime, performance of children in these ECE settings varied a lot.

Drawing on a dataset of 9,839 pupils at 136 primary schools from three less-developed provinces in Northwestern China, this paper seeks to investigate whether attending preschool or/and kindergarten promotes the cognitive and non-cognitive skills of pupils. Pupils’ cognitive skills are measured by the Third International Mathematics and Science Study (TIMSS) test and grade retention, while their non-cognitive skills are measured by self-efficacy, mental health and behaviors observed by their homeroom teachers. We first present skills, personal and family characteristics of pupils by ECE type and their differences. Further, to address the potential self-selection bias of attending ECE, we employ the Inverse Probability Weighting (IPW) techniques to balance the pre-treatment variables between groups, where we compare the Any ECE, Preschool Only, Kindergarten Only, and Preschool+Kindergarten group to No ECE group (the comparison group), respectively. Further, we employ the heterogeneous analyses to explore whether disadvantaged pupils benefit more from different types of ECE than their advantaged peers. Finally, from the perspective of curriculum contents, pedagogy in the classroom, qualification of teachers, exposure years, etc., we discuss the differences between kindergarten and preschool in rural China to explore the potential reasons for their differential effects on pupils.

Our paper contributes to at least three strands of literature. First, to the best of our knowledge, this study is one of the first to evaluate the benefits of different types of ECE, preschool and kindergarten, respectively in developing countries. To date, some of the relevant studies focusing on developed countries have revealed that the benefits of ECE differ by type. For instance, for the center-based care and Head Start in the United States, their effects on promoting children’s cognitive and social development varied widely [19–22]. Still the evidence in Denmark suggested that children attending center-based preschool performed better than those attending family daycare [23]. However, knowledge is limited about the heterogenous benefits of preschool and kindergarten except for the findings of Rao et al. [24] in southwestern China, which revealed that children attending preschool differed in mathematics and literacy scores from those attending kindergarten in grade one. Through the cross-provincial data with a large sample, this paper provides empirical evidence of the benefits of different types of ECE in a representative developing country and sheds light on the policies to expand effective type of ECE programs.

Moreover, our findings add to a growing body of literature on the long-term return to universal ECE in developing countries by providing supportive evidence for the ECE benefits in rural China. There is a growing evidence that universal ECE improves children’s academic performance [10,25–27], language, cognitive, and social-emotional ability [5,16,28,29] in primary or middle school, reduces their probability of dropping out [6] and leads to higher educational attainment [4,6]. Evidence focusing on the whole China also suggests positive cognitive and non-cognitive benefits for junior high students [28,30]. However, the long-term benefits of ECE in rural China are under-researched and the relevant results are mixed. Some studies suggest no impact on school readiness of Grade One pupils in Northern China [31], while some show that ECE experience is unrelated to cognitive skills but positively related to social skills of children aged 11–15 years old [9]. This paper adds to relevant literature by showing that specific type of ECE not only provides cognitive benefits, but also correlates to better behavioral performance of rural pupils.

Third, our study draws on more precise observational data reported by teachers to measure pupils’ behaviors, further adding to the literature relevant to the non-cognitive benefits of ECE in the context of developing countries. As to the findings of Feng et al. [32], compared with self-reported or parental-reported non-cognitive performance, teacher reports have the highest internal consistency and are the most predictive of children’s later behaviors in school. However, in the few studies focusing on the non-cognitive benefits of ECE in developing countries, children’s non-cognitive skills are typically measured using self-reported psychological scales [16,29], social behaviors and even the number of friends [9,28], or caregiver-reported social competence [5]. Considering that the self-reported non-cognitive skills are sensitive to survey conditions, the effects of interventions on self-reported noncognitive skills should be interpreted with caution [32,33]. Thus, this paper contributes to the literature with the help of homeroom teachers’ observation and recording of pupils’ performance in the classroom for a month on average, and our results further emphasize the importance of scientific measurement of non-cognitive abilities.

The rest of this paper is organized as follows. The next section introduces the characteristics of two main types of ECE, kindergarten and preschool, in rural China. Section 3 describes our data and analytical strategies. Section 4 presents our main results, and we discuss our results further in Section 5. Section 6 concludes.

## Preschool and kindergarten in rural china

The ECE system for 3- to 6- year-olds in rural China can be divided into two types: preschool and kindergarten. Preschool provides no more than three years of education through private-owned or community-owned centers [24,34]. Kindergarten (or pre-primary classes), typically affiliated with, and operated by rural primary schools, is usually the one- or two-year ECE program before children enter the first grade [12,18]. Generally, they differ a lot in training goals and teaching modes. The overall goal of preschool is to promote child development in the following five areas: behavior habits, movement ability, physical and mental health, intelligence, morality and art, while kindergarten has the additional goal of promoting children’s school readiness [35]. Such being the case, curriculums in rural preschools are often play-based, whereas those in kindergartens are relatively more academic [24]. Also, group teaching modes and Grade One syllabus (including literacy and numeracy) are more often used in kindergarten rather than that in preschool [12].

The coexistence of preschool and kindergarten in the ECE system has a long history in rural China. Preschool, which is the primary form of ECE program in urban areas and also present in rural areas, was first inaugurated in the early-1950s in rural areas [36]. During the same period, the prototype of kindergarten, which was named as “Nong Mang Child-Care Center”, emerged in rural villages to relieve mothers from childcare to agricultural production [36]. In 1983, the State Education Commission of China first stipulated that rural ECE could take various forms, including independent preschools or kindergartens affiliated to primary schools, and kindergarten was regarded as the major form of developing rural ECE [35]. The importance of the two types of ECE programs was further documented by the State Education Commission in 1991 [35].

Although rural China has witnessed great progress in the enrollment of formal preschool in recent years, the importance of kindergarten persists, especially in undeveloped western rural areas. Due to the lack of preschools, kindergarten once dominated rural ECE in the 1980s-1990s [37]. By 2008, the proportion of kindergarten enrollment had increased to 52% in the whole ECE enrollment in rural China [38]. During the same period, a survey of Shaanxi province (conducted by the authors before the survey in 2009, which covered 64 primary schools randomly selected) showed that nearly 60% of poor rural primary schools owned affiliated kindergartens, while less than 17% owned preschools. Despite the expansion of rural preschools since 2010 (from 113,000 in 2011 to 192,000 in 2019) [39], some western provinces still document the necessity for constructing kindergarten in remote rural areas, such as Qinghai and Ningxia [40,41].

Based on the complementarity and substitutability of preschool and kindergarten, there are three types of ECE choice available for rural children. On the one hand, preschool and kindergarten are complementary in teaching contents to some extent, which gives rural families the chance to send their children to preschool first to play with peers and then to kindergarten to prepare for primary school studies. On the other hand, similar attributes of the two ECE programs (e.g., both are center-based childcare agencies) lead some rural families to choose only one of them. Meanwhile, many rural areas have only one type of the ECE programs, or even neither. Children in remote rural areas therefore may only attend the ECE program to which they have access. To conclude, the counterfactual to no ECE experience in rural China includes preschool only, kindergarten only, and both preschool and kindergarten. However, to the best of our knowledge, little has been known about which ECE experience is the most effective.

## Materials and methods

### Sampling

The data used in this study come from two different projects that happened to collect the same information around the same time in three provinces of northwestern China, Shaanxi, Qinghai, and Ningxia, in 2009. They were collected in the baseline survey of a nutrition intervention program named Nutrition and Education, which was initiated by the REAP (Rural Education Action Program) team. Scholars from many universities, including Stanford University, Peking University, Shaanxi Normal University, Xi’an Jiaotong University, and so on worked together for this program. Conducting the study in these provinces is advantageous due to their representation of undeveloped areas of China. In 2009, the average per-capita income of rural residents in Shaanxi, Qinghai, and Ningxia were RMB 3722 (USD 558), 3477 (USD 522) and 4405 (USD 661), respectively, much lower than the national average of RMB 5435 (USD 815) [42]. To be specific, nationally designated poverty counties (in 2014) accounted for more than 30% in all three provinces, and even more than 95% in Qinghai [43].

In the above three provinces, 10,804 pupils selected by the random-sampling procedure participated in the survey, and 9,839 of them both reported their ECE experiences and finished skill tests became our final study sample. To be specific, data used in this study were pooled from two different projects in Shaanxi and Qinghai & Ningxia that happened to collect the same information around the same time. The difference lies mainly in the way we selected our sample counties within the province. In Shaanxi, the project team randomly selected ten counties with a majority population in rural areas as sample counties. In Qinghai and Ningxia, five nationally designated poverty counties were randomly selected as sample countries in each province. As Shaanxi was more populous (ranking the 19th out of 31 provinces in 2009) than Qinghai and Ningxia (ranking the 29th and 30th, respectively), the project team selected more countries in Shaanxi than that in Qinghai and Ningxia. Also, as Shaanxi was more developed (ranking the 17th out of 31 provinces in total GDP in 2009) than Qinghai and Ningxia (ranking the 29th and 30th, respectively) with a higher level of urbanization, in Shaanxi, counties with a majority population in rural areas were used to select sample counties, while in Qinghai and Ningxia, the list of the nationally designated poverty counties was used to select sample countries.

Another difference is in the way we constructed the sampling frame of schools within counties. In Shaanxi, schools in the sampling frame had to meet the following two criteria simultaneously: having an enrollment greater than 200 students and having at least 50 boarding pupils. In Qinghai and Ningxia, however, the inclusion criteria was slightly different: having an enrollment greater than 400 pupils and boarding facilities. After selecting sample counties and schools, all the grade four pupils in Shaanxi and all the grade four and five pupils in Qinghai and Ningxia in our sample schools were surveyed. Finally, those (9,839) both reporting their ECE experiences and finishing skill tests were used as our study sample.

For the purpose of this study, we draw on information from six modules. The first module is the student survey where we collected information on pupils’ demographic characteristics (gender, age, ethnicity, number of siblings, etc.), their ECE experience, self-efficacy and mental health. The sample pupils were also administered a 30-minute math test followed by the survey, including 30 questions selected from the Trends in International Mathematics and Science Study (TIMSS) test data bank. The third is the household survey including parental demographic information (age, years of schooling, migrating status, etc.), availability of household assets and so on. Fourth, Chinese and Mathematical teachers reported their education, experience, professional ranks and awards in the teacher questionnaires, and then provided us with the Chinese and Math scores of pupils in the final exams of the fall semester in 2009. We also collected information on primary school quality through the principal questionnaire, including qualifications of the principal and teachers, class size and school facilities. In addition to the household questionnaire completed by parents at home and collected by the homeroom teachers, other questionnaires were filled out by respondents under the guidance of trained enumerators in the survey school.

Besides the first five modules collected during the survey, there is also a module collecting information observed by teachers after the survey. Homeroom teachers of the sample pupils were asked to complete a short survey form to evaluate their in-school behaviors in the few weeks following the survey. To be specific, they recorded the number of times when students had the following behavioral problems (from Monday to Friday): being late or leaving early, skipping the class, being absentminded in the class. On average, the classroom performance of these pupils was recorded for an average of four weeks, which allows us to examine the pupils’ behavioral problems drawing on observational data over a long period.

### Variables

Our analyses focus on two cognitive outcomes and three non-cognitive outcomes. For cognitive outcomes, we first use the standardized scores on TIMSS test to create a measure of “TIMSS score”. Also, the grade retention is defined as a binary indicator that equals one if the pupil has ever retained in primary school and zero otherwise. Non-cognitive outcomes include both the self- and teacher-reported indicators. In terms of self-reported outcomes, we use the Chinese version of general self-efficacy scale to measure their self-efficacy, which includes 10 questions on a four-point scale and ranges from 10–40, where the higher score indicates higher non-cognitive skills. We also use the Mental Health Test (“MHT”, 100 yes/no questions, ranging from 0–100) to measure pupils’ mental health, which was developed by Zhou [44]. To check the reliability of self-efficacy and MHT, we calculated their Cronbach’s alpha coefficients, and the results show that the Cronbach’s alpha coefficients are 0.638 for self-efficacy and 0.745–0.909 for MHT, respectively, suggesting both measures have acceptable reliability in our study sample [45]. As to teacher-reported behavioral problems, the principal component analysis (PCA) was used to create a composite score combining the measures of the three kinds of behavioral problems (No. of skipping classes per week, No. of being late or leaving early per week, No. of being absentminded in the class per week) and then standardize the score, where the higher score indicates more behavioral problems.

We measure the ECE experience with one dummy variable and the ECE type with three dummy variables. The student survey has asked two relevant questions, the first is “Whether you have attended preschool?” and the second is “Whether you have attended kindergarten?”. The dummy variable “ECE attendance” equals one if the answers are not zero for at least one question and equals zero otherwise. Then three mutually exclusive dummy variables are used to distinguish ECE types, where “Preschool only” equals one if the answer is not zero to the first question but zero to the second, “Kindergarten only” equals one if the answer is zero to the first question but not zero to the second, “Preschool+kindergarten” equals one if the answer is not zero to both the questions, otherwise these variables take the value of zero.

Following the literature, we control for characteristics at the student, parent and household levels that might affect pupils’ development outcomes. Specifically, we control for five covariates at the student level (including age, gender, ethnicity, boarding status, number of siblings), five covariates at the parent level (including age of parents, years of schooling of parents, parents outside home), and household asset index at the household level. Following Wong et al. [31], we took a three-step procedure to construct a household asset index to measure family social economic status (SES). First, we counted how many types of the 21 types of fixed assets are owned by a household, with 21 indicating having all types whereas zero none. The 21 types of fixed assets include any bicycle, motorbike, electric bicycle, tractor, car, van, telephone, mobile phone, video, audio system, color TV, VCD/DVD, gas stove, microwave ovens, refrigerator, camera, video camera, computer, electric fan, air conditioner, and laundry machine. In the second step, we used the PCA method to get a composite score. Finally, we standardized the score to get the household asset index, which ranged between -2.533 and 11.022.

### Ethics statement

In designing and implementing this study, we paid close attention to the ethical concerns that arose during our survey. Due to that the program brong together scholars from more than one country, the implementation plan of the program was reported to and approved by the ethics committees from both China and the United States respectively. To be specific, our program obtained ethical approval from the Institutional Review Board (IRB) of the Stanford University (Protocol ID: 19748) and the Xi’an Jiaotong University (Protocol ID: 00003556). In accordance with the IRB requirements, the study pupils provided oral assent for the project, and the school principals, who were the pupils’ legal guardians while they were in school, provided their written consents. Their consents were recorded and collected by the interviewers. All the students received letters informing their parents of their health status. All the students found to have mental health or behavioral problems received treatment in the classroom. After the data cleaning and matching, all the potential identifying information was removed by the authors.

### Empirical strategy

To identify the benefits of any ECE experience on pupils’ skill development, we divide our study sample into two groups: any ECE and No ECE. Within any ECE group, we further differentiate three groups: Preschool Only, Kindergarten Only, and Preschool+Kindergarten. We use the following empirical specification to identify the impact of any ECE experience on pupils cognitive and non-cognitive skills:

$$Y_{ic}=\beta_{0}+\beta_{1}AnyECE_{ic}+X_{ic}\beta +\gamma_{c}+\varepsilon_{ic}$$ (1)

Where *Y*_*ic*_ denotes the cognitive and non-cognitive outcomes of student *i* in class *c*. *AnyECE*_*ic*_ is a dummy variable taking the value of one if a pupil belongs to Any ECE group and zero if the pupil comes from the No ECE group. ***X***_***ic***_ denotes a set of covariates including individual characteristics (age, gender, ethnicity, boarding status, number of siblings), parental characteristics (age and years of schooling of parents and parents outside home) and household socioeconomic status (household asset index). *γ*_*c*_ denotes the classroom fixed effects, which are used to control for characteristics affecting student achievement at the class level and above *ε*_*ic*_ is the i.i.d. error term. Standard errors are clustered at the class level.

When we replace the Any ECE dummy in Eq (1) with the dummy of Preschool Only group, Kindergarten Only group, or Preschool+Kindergarten group one at a time in turn, we will be able to identify the impact of these three different types of ECE experience on pupils’ cognitive and non-cognitive outcomes, respectively.

**Fig 1:**
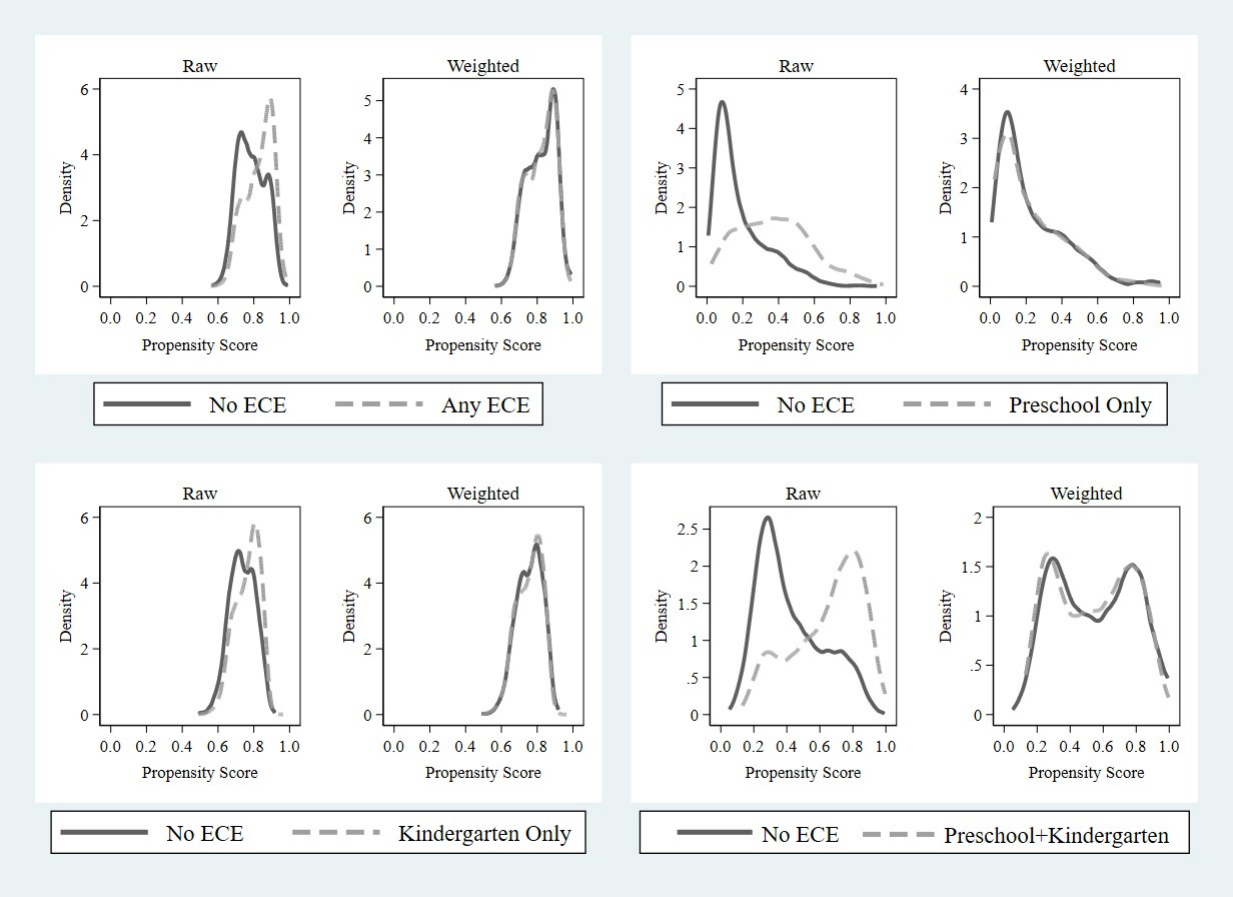
Diagnostic diagrams of IPW.

In estimating Eq (1), IPW is used to balance the pre-treatment variables between groups in order to alleviate the potential selection bias. Taking Any ECE group as an example, this is how we implement it in three steps. In the first step, we use the Stata command “*psestimate*” proposed by Imbens and Rubin [46] to select a quadratic function of covariates *X*_*ic*_ for the estimation to calculate the propensity score a sample pupil would have any ECE experience. In the second step, the propensity scores are used to assign the treatment sample (those with any ECE experience) a weight of one whereas the comparison sample (those with no ECE experience) a weight equal to the predicted odds of being in a treatment case (ρi/(1-ρi)). Finally, we bring the weights into Eq (1) and calculate the weighted estimators. Fig 1 provides the density plots of propensity scores before and after re-weighting, which demonstrates well-balanced groups post-weighting.

We then conduct a three-step PSM approach as a robustness check of results from IPW. Taking the Any ECE group as an example, in the first step, we check whether there is a large overlap in the support of the covariates between the treatment and comparison groups. Our data show that the common support is fairly wide, demonstrating the large overlap in the propensity scores. In the second step, we use the radius matching, local linear regression matching and kernel matching, respectively, to construct a counterfactual match for the any ECE group based on the PSM. Finally, we assess the matching quality by results from the balance test. We find that the matching procedure does a good job of balancing the distribution of the relevant covariates in both the comparison (No ECE) and treatment (Any ECE) groups.

## Results

### Characteristics of the sample pupils

**Fig 2:**
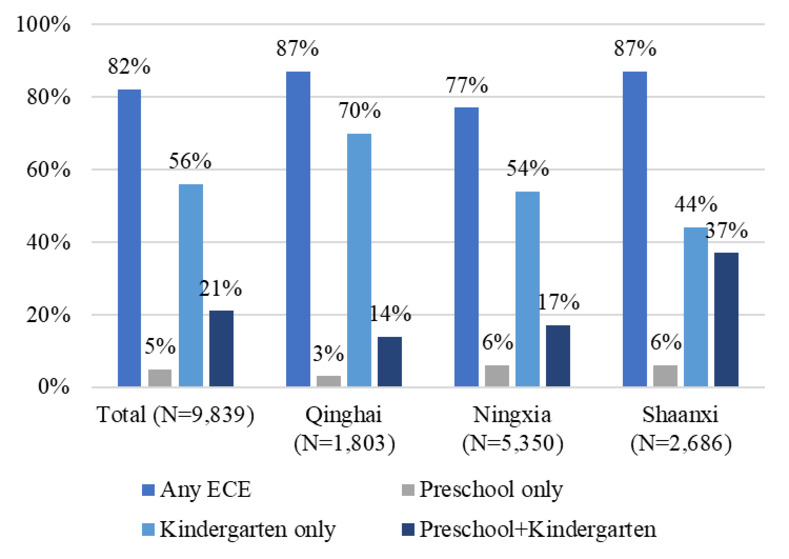
ECE enrollment rate by provinces.

According to our data, the ECE enrollment of the sample pupils is as high as 82% with the majority attending kindergarten only (Fig 2). Specifically, among those with ECE experience, 56% ever went to kindergarten only, 5% went to preschool only, and 21% went to both preschool and kindergarten. The enrollment rate also varies across three provinces. It is found that the overall ECE enrollment rate in Shaanxi and Qinghai is 11 percentage points higher than that in Ningxia. Further, by ECE type, Shaanxi has the highest proportion of pupils attending both preschool and kindergarten (35%), while Qinghai has the highest proportion of the sample pupils attending kindergarten only (72%) and the lowest proportion of those attending preschool only (3%).

Descriptive statistics show that pupils’ cognitive and non-cognitive skills vary by ECE type (Table 1). In terms of cognitive skills, pupils with ECE experience perform much better in the TIMSS than their peers without any ECE experience, with the Peschool+Kindergarten group scoring the highest (0.13 SD), outscoring the No ECE group significantly by nearly 0.32 SD. Meanwhile, the Preschool Only group and the Kindergarten Only group outscore the No ECE group in the TIMSS by 0.21 and 0.23 SD, respectively. A similar pattern appears when we look at the likelihood of grade retention, with those who attended both preschool and kindergarten being 12.0 percentage points less likely ever to retain the grade than those without any ECE experience. As to non-cognitive skills, different patterns emerge. In terms of self-efficacy and behavioral problems, pupils’ performance differs little by ECE type. However, compared to pupils without any ECE experience, those in the Preschool Only, Kindergarten Only and Preschool+Kindergarten group score 8.7, 3.4 and 13.7 points less in the MHT, respectively, suggesting that those with ECE experience perform significantly better in mental health than their peers without ECE experience.

**Table 1:** Cognitive and non-cognitive skills of the sample pupils, by different ECE types.

	No ECE	Any ECE	Preschool only	Kindergarten only	Preschool+ kindergarten	Diff (2)-(1)	Diff (3)-(1)	Diff (4)-(1)	Diff (5)-(1)
	(1)	(2)	(3)	(4)	(5)	(6)	(7)	(8)	(9)
TIMSS	-0.182	0.065	0.025	0.043	0.133	0.247***	0.207***	0.225***	0.315***
Grade retention	0.495	0.385	0.377	0.390	0.375	-0.110***	-0.118***	-0.105***	-0.120***
Self-efficacy	25.35	25.46	25.49	25.42	25.56	0.107	0.135	0.064	0.211
MHT score	35.83	29.37	27.16	32.42	22.09	-6.459***	-8.669***	-3.404***	-13.731***
Behavioral problems	-0.046	-0.009	-0.055	0.007	-0.040	0.037	-0.009	0.053	0.006

Notes

^a^ ‘Diff’ is the difference in means of the variables for the sample pupils with or without ECE experience, where *** p < 0.01, ** p < 0.05, * p < 0.1.

^b^ Source: Authors’ survey.

When we compare the basic characteristics of pupils by ECE type, our data show a broad barry of individual and family characteristics are associated with pupils’ ECE experience (Table 2). Specifically, younger pupils are more likely to have ECE experience. Moreover, conditional on those with ECE experience, younger pupils are also more likely to attend preschool than kindergarten. Boarders, ethnic minorities and those with more siblings and older parents are less likely to have ECE experience than their peers. In contrast, pupils with more educated parents, migrant parents and higher household asset index are more likely to have ECE experience.

**Table 2:** Individual and household characteristics of the sample pupils, by different ECE types.

	No ECE	Any ECE	Preschool only	Kindergarten only	Preschool+ Kindergarten	Diff (2)-(1)	Diff (3)-(1)	Diff (4)-(1)	Diff (5)-(1)
	(1)	(2)	(3)	(4)	(5)	(6)	(7)	(8)	(9)
Female	0.483	0.475	0.468	0.474	0.480	-0.008	-0.015	-0.009	-0.003
Age	10.89	10.65	10.31	10.82	10.29	-0.243***	-0.587***	-0.071**	-0.606***
Ethnic minority	0.611	0.406	0.270	0.479	0.249	-0.205***	-0.341***	-0.132***	-0.362***
Boarder	0.377	0.336	0.192	0.383	0.247	-0.041***	-0.184***	0.007	-0.130***
Numbers of siblings	2.507	2.093	1.857	2.208	1.851	-0.414***	-0.651***	-0.299***	-0.656***
Father’s age	38.67	38.30	38.01	38.38	38.17	-0.369***	-0.657***	-0.292**	-0.497***
Mother’s age	35.92	35.57	35.52	35.60	35.48	-0.357***	-0.407*	-0.319***	-0.443***
Father’s years of schooling	3.695	5.170	6.125	4.542	6.569	1.475***	2.430***	0.848***	2.875***
Mother’s years of schooling	2.075	3.205	4.596	2.457	4.808	1.129***	2.521***	0.382***	2.733***
Parents outside home	0.373	0.407	0.389	0.397	0.438	0.035***	0.016	0.025*	0.065***
Household asset index	-0.301	-0.027	0.594	-0.259	0.421	0.274***	0.895***	0.042	0.722***

Notes

^a^ ‘Diff’ is the difference in means of the variables for the sample pupils with or without ECE experience, where *** p < 0.01, ** p < 0.05, * p < 0.1.

^b^ Source: Authors’ survey.

### ECE attendance and pupils’ cognitive and non-cognitive skills

So far, our results from IPW have shown that ECE experience benefits pupils’ cognitive skills but not their non-cognitive skills (Table 3, Panel A). Compared with their peers without any ECE experience, pupils with any ECE experience perform better in TIMSS by 0.118 standard deviations (s.d.) and are 7.1 percentage points (pp) less likely to ever retain a grade, both are significant at the 1% level. However, pupils with any ECE experience differ little in non-cognitive skills from their peers with no ECE experience.

**Table 3:** ECE experience and cognitive/non-cognitive outcomes of pupils.

	TIMSS	Grade retention	GSES	MHT score	Behavioral problems
	(1)	(2)	(3)	(4)	(5)
***Panel A*: *Any ECE V*.*S*. *No ECE***					
Any ECE	0.118***	-0.071***	0.112	-0.441	-0.045
	(0.025)	(0.015)	(0.161)	(0.341)	(0.029)
Observations	9,839	9,839	9,839	8,871	9,839
R-squared	0.218	0.214	0.056	0.754	0.588
***Panel B*: *Preschool Only V*.*S*. *No ECE***					
Preschool Only	-0.080	-0.024	0.002	0.016	0.004
	(0.070)	(0.037)	(0.390)	(0.833)	(0.059)
Observations	2,288	2,288	2,288	2,027	2,288
R-squared	0.380	0.283	0.177	0.748	0.444
***Panel C*: *Kindergarten Only V*.*S*. *No ECE***					
Kindergarten Only	0.150***	-0.070***	0.040	-0.272	-0.059*
	(0.026)	(0.015)	(0.167)	(0.358)	(0.035)
Observations	7,222	7,222	7,222	6,463	7,222
R-squared	0.219	0.224	0.065	0.708	0.588
***Panel D*: *Preschool+Kindergarten V*.*S*. *No ECE***					
Preschool+kindergarten	0.035	-0.045**	0.243	-0.823	-0.039
	(0.040)	(0.021)	(0.210)	(0.524)	(0.034)
Observations	3,845	3,845	3,845	3,459	3,845
R-squared	0.296	0.267	0.133	0.808	0.497
Control	YES	YES	YES	YES	YES
Class FE	YES	YES	YES	YES	YES

Notes

^a^ Standard errors clustered at the class level appear in parentheses. *** p < 0.01, ** p < 0.05, * p < 0.1.

^b^ Reference group is the No ECE group.

^c^ Control variables include female, age, ethnic minority, boarder, numbers of siblings, father’s age, mother’s age, father’s years of schooling, mother’s years of schooling, parents outside home and household asset index.

We then turn to the benefits by ECE type on pupils’ development. Results from IPW show that compared to their peers without any ECE experience, pupils attending preschool only do not perform better in cognitive or non-cognitive outcomes (Table 3, Panel B). In contrast, pupils attending kindergarten only perform better in TIMSS (0.150 s.d. higher) and behavioral problems (0.059 s.d. lower), as well as 7.0 pp less likely to ever retain a grade (Table 3, Panel C). Moreover, pupils attending both preschool and kindergarten are less likely to have retained any grade (by 4.5 pp), but have little impact on non-cognitive outcomes (Table 3, Panel D). Overall, our results show that the cognitive and non-cognitive returns to ECE attendance are mainly driven by attending kindergarten only.

### Heterogeneous analyses

Since we have demonstrated that attending kindergarten rather than preschool can predict pupils’ cognitive and non-cognitive skills, one question naturally arises: whether disadvantaged children benefit more from ECE attendance than their advantaged peers? If so, which type of ECE benefits disadvantaged pupils’ cognitive and non-cognitive development more? Answering this question helps with improving the understanding of benefits of ECE attendance, and has implications for the ECE policy in rural areas. To be specific, we focus on three kinds of disadvantaged children: pupils from families with relatively lower SES, girls and ethnic minorities. We generate the corresponding three dummy variables, high ESE (= 1 if the household asset index exceeds the median), girl (Yes = 1) and ethnic minorities (Yes = 1), then introduce the aforementioned dummy variables into the IPW approach and interact it with the indicators of ECE type before we reran the regressions (in Tables 4–6).

**Table 4:** ECE types and cognitive/non-cognitive outcomes of pupils, by household asset index.

	TIMSS	Grade retention	GSES	MHT	Behavioral problems
	(1)	(2)	(3)	(4)	(5)
***Panel A*:**					
Preschool only ×High SES	0.106	0.024	0.320	0.122	-0.123
	(0.119)	(0.053)	(0.690)	(1.789)	(0.092)
Observations	2,282	2,282	2,282	2,021	2,282
R-squared	0.404	0.371	0.206	0.817	0.557
***Panel B*:**					
Kindergarten only×High SES	0.039	0.025	0.556*	-0.747	-0.083
	(0.056)	(0.028)	(0.309)	(0.649)	(0.054)
Observations	7,219	7,219	7,219	6,461	7,219
R-squared	0.248	0.236	0.082	0.723	0.534
***Panel C*:**					
Preschool+kindergarten×High SES	0.013	0.005	0.380	-1.071	-0.102*
	(0.086)	(0.040)	(0.475)	(0.847)	(0.053)
Observations	3,835	3,835	3,835	3,450	3,835
R-squared	0.280	0.289	0.115	0.841	0.492
Control	YES	YES	YES	YES	YES
Class FE	YES	YES	YES	YES	YES

Notes

^a^ IPW method is used in all the regressions.

^b^ All the regressions include class fixed effects, and standard errors clustered at the class level appear in parentheses. *** p < 0.01, ** p < 0.05, * p < 0.1.

^c^ Reference group is the No ECE group.

^d^ Control variables include female, age, ethnic minority, boarder, numbers of siblings, father’s age, mother’s age, father’s years of schooling, mother’s years of schooling, parents outside home and household asset index.

**Table 5:** ECE types and cognitive/non-cognitive outcomes of pupils, by gender.

	TIMSS	Grade retention	GSES	MHT	Behavioral problems
	(1)	(2)	(3)	(4)	(5)
***Panel A*:**					
Preschool only ×Girl	-0.069	0.092	0.055	-1.504	-0.020
	(0.117)	(0.060)	(0.595)	(1.271)	(0.074)
Observations	2,282	2,282	2,282	2,021	2,282
R-squared	0.408	0.371	0.207	0.818	0.556
***Panel B*:**					
Kindergarten only ×Girl	-0.004	-0.015	0.333	0.921	-0.014
	(0.048)	(0.026)	(0.295)	(0.638)	(0.049)
Observations	7,219	7,219	7,219	6,461	7,219
R-squared	0.250	0.236	0.082	0.723	0.534
***Panel C*:**					
Preschool+kindergarten×Girl	0.080	0.020	0.092	-1.231	-0.082
	(0.079)	(0.038)	(0.427)	(0.847)	(0.060)
Observations	3,835	3,835	3,835	3,450	3,835
R-squared	0.283	0.288	0.115	0.841	0.492
Control	YES	YES	YES	YES	YES
Class FE	YES	YES	YES	YES	YES

Notes

^a^ IPW method is used in all the regressions.

^b^ All the regressions include class fixed effects, and standard errors clustered at the class level appear in parentheses. *** p < 0.01, ** p < 0.05, * p < 0.1.

^c^ Reference group is the no ECE group.

^d^ Control variables include female, age, ethnic minority, boarder, numbers of siblings, father’s age, mother’s age, father’s years of schooling, mother’s years of schooling, parents outside home and household asset index.

**Table 6:** ECE types and cognitive/non-cognitive outcomes of pupils, by ethnicity.

	TIMSS	Grade retention	GSES	MHT	Behavioral problems
	(1)	(2)	(3)	(4)	(5)
***Panel A*:**					
Preschool only ×Ethnic minority	0.070	0.056	-0.622	1.086	0.161
	(0.135)	(0.066)	(0.761)	(1.626)	(0.112)
Observations	2,282	2,282	2,282	2,021	2,282
R-squared	0.408	0.370	0.207	0.818	0.557
***Panel B*:**					
Kindergarten only ×Ethnic minority	-0.026	0.001	0.146	-0.122	0.049
	(0.054)	(0.030)	(0.347)	(0.737)	(0.049)
Observations	7,219	7,219	7,219	6,461	7,219
R-squared	0.250	0.236	0.081	0.723	0.534
***Panel C*:**					
Preschool+kindergarten× Ethnic minority	-0.074	0.053	0.190	-1.125	0.101*
	(0.079)	(0.042)	(0.451)	(0.982)	(0.058)
Observations	3,835	3,835	3,835	3,450	3,835
R-squared	0.283	0.288	0.115	0.841	0.492
Control	YES	YES	YES	YES	YES
Class FE	YES	YES	YES	YES	YES

Notes

^a^ IPW method is used in all the regressions.

^b^ All the regressions include class fixed effects, and standard errors clustered at the class level appear in parentheses. *** p < 0.01, ** p < 0.05, * p < 0.1.

^c^ Reference group is the no ECE group.

^d^ Control variables include female, age, ethnic minority, boarder, numbers of siblings, father’s age, mother’s age, father’s years of schooling, mother’s years of schooling, parents outside home and household asset index.

Our heterogenous analyses first show that pupils from higher-SES families benefit more from attending ECE than their counterparts (Table 4). Considering the results for self-efficacy, point estimates suggest a larger association with attending kindergarten only for the high-SES group than their low-SES peers. As to behavioral problems, those from better-off families benefit more from attending both preschool and kindergarten compared to their disadvantaged counterparts.

We then find that the benefits for girls do not differ from that for boys (Table 5), while the Han pupils benefit more from ECE experience than their counterparts (Table 6). Specifically, Table 6 reveals a stronger positive relationship between attending both preschool and kindergarten and pupils’ behavioral problems among ethnic minorities than their peers. Besides, there are no significant gender and ethnic differences. To sum up, we find that ECE attendance is not necessarily more beneficial to disadvantaged children. It seems that inequalities in the ECE benefits among pupils of different socioeconomic status and ethnic groups emerge to some extent.

### Robustness check

To verify the robustness of our results, we replace IPW techniques with the PSM methods, including radius matching, local linear regression matching and kernel matching, respectively. The results of these alternative approaches are almost identical to those previously described, which further support the robustness of our IPW estimates.

As Inverse Probability Weighting (IPW) controls for observed characteristics only, we still need to take care of the potential unobserved differences between the treatment and the comparison groups. To assess whether omitted variable bias might have driven our results, we follow Oster [47] by calculating the ratio of selection on unobserved characteristics over selection on observed characteristics. According to Oster [47], a positive ratio greater than one suggests that for the selection on unobserved characteristics to fully explain the estimated effects, it must be greater than that on observed characteristics. This is unlikely to be the case when many covariates are controlled. In contrast, a negative ratio suggests that the estimated effects are biased downward and that adding more controls could make the coefficient larger. Our data show that regardless of the dependent variables, the Oster ratios turn out to be much greater than 100, suggesting it is unlikely that our results are driven by unobserved differences between the treatment and the comparison groups, namely, those with and without ECE experience.

There is also a concern with our results that the selection on school quality and non-random classroom assignment during the period of primary school, if it exists, might account for inconsistent benefits of different types of ECE. Skills accumulated in the early life are only likely to persist if subsequent school or classroom environments benefit students continually [21,22]. If children with kindergarten experience are more likely to enroll in higher-quality primary schools or to be assigned with better teachers than those with preschool experience, the heterogeneous effects of different ECE types are not due to their own quality differences, but to the difference in the primary school quality. To examine the possibility, we examine the relationship between types of ECE attended by pupils and their primary school quality (indicated by qualifications of principals, class size, the average rank of teachers and availability of equipment) as well as the class quality (indicated by qualifications of Chinese and Math teachers), and find that majority of the coefficients come out not significant in addition to four inconsistent coefficients. In other words, pupils attending different kinds of ECE differ little in the educational resources available at the primary school level. Thus, we can conclude that primary school quality does not shape the benefits of ECE attendance.

## Discussion

### Comparison with previous studies

Our data show that the grade retention rate of pupils in the No ECE and Any ECE groups reaches 50% and 39%, respectively, much higher than the findings of 24.6–32.4% in Oklahoma in the United States [22]. Moreover, parents in most groups have an average of six years of school, indicating they have barely finished primary school education. We also find children from better-off families are more likely to have ECE experience and to attend both preschool and kindergarten. This finding is consistent with that of Coley et al. [19] among low-income children in the U.S., where they find children from better-off families are not only more likely to attend ECE, but also more likely to attend private ECE programs.

Our estimates of the benefits of ECE experience in rural China differ from those reported in the literature. While some studies using the China Education Panel Survey (CEPS) data in China found both cognitive and non-cognitive benefits associated with ECE experience [28], we find that in rural northwestern China, ECE experience seems to exert cognitive benefits only. Moreover, the magnitude of benefits on TIMSS in this study is only 66–74% of that reported in the literature using CEPS data (0.159–0.180 s.d.) [28,30]. As Zheng et al. [30] and Zhang [28] found that the cognitive benefits of ECE decline with age among middle school students and the cognitive benefits of ECE decline with age [48], we might expect the actual difference in the magnitude between our estimates and those reported in the literature to be even larger. Lastly, our results are in contrast to those of Gong et al. [9] who found in rural China that the benefits of ECE experience are reflected in non-cognitive rather than cognitive skills.

Nonetheless, the different benefits by ECE type that we have found in this study are consistent with several previous studies. For instance, it has been found in both the U.S. and Denmark that center-based care differs greatly from other ECE types in promoting child development [19,49]. Also, Rao et al. [24] drew on a small sample of 207 pupils in a southwestern county in China and found that the cognitive performance of pupils attending preschool or kindergartens differed.

In terms of heterogeneous effects, we find that the benefits of ECE are more pronounced among the Han pupils from higher SES families. Our findings that the little difference between ECE benefits of girls and boys are not consistent with those of Blanden et al. [50] and Bietenbeck et al. [10]. Also, our findings that pupils from higher SES families benefit more differ from some previous studies [5,10,28,50,51]. One of the possible explanations behind the inconsistency is that the quality varies more among preschools than kindergartens. This is not surprising considering the fact that private preschools once dominated the rural areas before 2010, as indicated by the wide variations in fees and quality among these private preschools [36]. More advantaged families, e.g., those with higher SES and the Han families, are more likely to afford higher-priced but better-quality preschools, which helps to explain why pupils from better-off families benefit more from the preschool experience.

### Why do the cognitive and non-cognitive benefits on pupils differ by ECE types?

So far, our results have shown that the benefits of ECE experience on pupils, including promoting cognitive skills and reducing behavioral problems, are mainly driven by attending kindergartens rather than by attending preschools. A natural question arises as to why kindergarten play its role while preschool does not in the context of rural China. We try to provide some insights by comparing four major characteristics between preschools and kindergartens.

### Curriculum

The reason why kindergartens benefit pupils’ cognitive skills but preschools do not may have a lot to do with their curricula. The curricula in kindergartens are more academic than those in preschools due to that the former focuses more on cognitive development than the latter. It has been observed in rural China that basic arithmetic and literacy are often taught in kindergarten [24], and some kindergartens even use the grade one syllabus directly [12,52]. Such highly academic kindergartens have also been found in some developing countries like Kenya and Zambia [10,53]. In Kenya, kindergarten children sit at desks and listen to their teachers in a classroom-like setting. Their curricula, while not standardized, emphasize the learning of basic numeracy and literacy skills via memorization and recitation [10]. The academic-oriented curriculum settings in kindergartens may give children a head start and make it easier for them to follow the primary school curriculum.

### Pedagogy

Compared to preschools, kindergartens’ pedagogy emphasizes behavioral norms more, which helps to explain our findings that pupils with kindergarten experience have fewer behavioral problems. Moreover, kindergartens are more likely to conduct collectivized teaching modes and enforce disciplines like primary schools [12,52]. Luo et al. [52] observed that in rural Central China, many kindergarten teachers were also teachers of the primary schools with which their kindergartens were affiliated. Such kindergarten teachers may tend to practice primary school discipline requirements in kindergarten classrooms, which might help explain why pupils with kindergarten experience have fewer behavioral problems than their peers without any ECE experience.

### Shortages and low qualifications of preschool teachers

Another reason behind different impacts by ECE type on pupils’ skills in rural China might have something to do with shortages and low qualifications of preschool teachers. Provincial-level statistics show that during the years when sample children attended preschools (namely between 2004 and 2006), rural preschools in Shaanxi, Qinghai and Ningxia were suffering from teacher shortages. For instance, the child-teacher ratio (36:1–71:1) in rural areas of our three study provinces lagged far behind the national average for rural areas (38:1–45:1), not to mention those for urban areas (18:1–19:1). Moreover, there is evidence that rural preschool teachers are less experienced, and less likely to have access to high-quality training compared to their urban peers [52,54]. Such being the case, the shortage of preschool teachers would contribute to inadequate care for each child, and those teachers might be less qualified or prepared than kindergarten teachers to support children’s development.

### Duration of ECE

In addition to curriculum, pedagogy and teachers, there is still another concern that the difference in duration between preschools and kindergartens might contribute to the difference in estimated effects on pupils’ skills by ECE type. In the context of rural China, preschool duration (no more than three years) is usually longer than that of kindergarten (one or two years) [9,12]. To deal with this concern, we examined the average number of years of ECE attendance for different ECE groups. Surprisingly, the average duration for the Preschool Only group (1.5 years) is just slightly longer than that for the Kindergarten Only group (1.3 years) (p-value = 0.000). In the meantime, the Preschool+Kindergarten group has a much longer duration (2.8 years on average) than the aforementioned two groups. Considering the slight difference in duration between the Preschool Only and Kindergarten Only groups, and our results that the benefits of attending preschool only are little but those of attending kindergarten only or both of them are positive, it seems that difference in durations of ECE would not drive the differential returns by ECE type.

### Implications

We can draw at least three implications from the research findings. First, our findings about the overall benefits of ECE attendance suggest that increasing access to quality ECE might be an effective instrument to improve pupils’ cognitive skills in less-developed rural China. Considering the fact that pupils from disadvantaged families are less likely to attend ECE, and even among those with preschool experience, the quality of preschools they attended might vary a lot with family backgrounds, more efforts should be made to ensure equal access to quality ECE for every preschool-aged children in rural China. Second, the finding that kindergartens benefit pupils more in their skills than other types of ECE implies that more focus should be given to kindergartens in rural and less developed areas of China. Meanwhile, the lack of impact of preschools on promoting pupils’ skill development might have to do with its low quality. Therefore, more attention should be paid to improving the quality of rural preschools to facilitate their roles in promoting children’s skills. Last but not least, our research findings also imply the importance of distinguishing ECE type when evaluating the benefits of ECE programs in developing countries in further research.

## Conclusions

In this paper, we provide new evidence of the benefits of attending kindergartens or/and preschools on pupils’ skill development, drawing on data from pupils in rural areas of three provinces in northwestern China. We find that pupils with ECE experience perform better in cognitive skills than their peers without any ECE experience. We further show that attending kindergarten only benefits pupils’ skills by increasing their TIMSS by 0.150 s.d., decreasing their likelihood of grade retention by 7.0 pp, and reducing their behavioral problem index by 0.059 s.d.. In the meantime, attending both preschool and kindergarten is associated with a lower probability of grade retention. In contrast, pupils who attended preschool only performed as well as their peers without any ECE experiences in cognitive and non-cognitive skills. Results from heterogeneous analyses show that pupils from families with higher SES and the Han pupils benefit more from ECE experience. These findings remain robust to alternative matching methods and sustain relevant tests.

We acknowledge at least three limitations of this study. First, our dependent variables are based on recall data of ECE attendance. If recall error exists and systematically depends on preschool or kindergarten attendance, it will bias our estimates. Second, our sample pupils were surveyed in primary schools, and we did not have the opportunity to collect information on the quality of the preschools or kindergartens they attended, which limits the discussion underlying our findings. Finally, our data were collected in 2009, which can only reflect the benefits of rural ECE before 2010. With increasing investment in rural ECE since 2010, there has been evidence that preschool quality in rural areas has improved a lot, although it still lags far behind its urban peers [55]. Thus, whether the quality of rural preschools has reached a certain threshold to promote child development is an interesting and important topic for future studies.

## References

[1] Marope PTM, Kaga Y, editors. Investing against evidence: the global state of early childhood care and education. Paris: UNESCO Publishing; 2015.

[2] Cunha F, Heckman J. The technology of skill formation. Am Econ Rev. 2007;97: 31–47. doi: 10.1257/aer.97.2.31

[3] Cunha F, Heckman JJ, Schennach SM. Estimating the technology of cognitive and noncognitive skill formation. Econometrica. 2010;78: 883–931. doi: 10.3982/ECTA6551 20563300 PMC2885826

[4] Berlinski S, Galiani S, Manacorda M. Giving children a better start: Preschool attendance and school-age profiles. J Public Econ. 2008;92: 1416–1440. doi: 10.1016/j.jpubeco.2007.10.007

[5] Brinkman SA, Hasan A, Jung H, Kinnell A, Pradhan M. The Impact of Expanding Access to Early Childhood Education Services in Rural Indonesia. J Labor Econ. 2017 [cited 25 May 2022]. doi: 10.1086/691278

[6] Krafft C. Increasing educational attainment in Egypt: The impact of early childhood care and education. Econ Educ Rev. 2015;46: 127–143. doi: 10.1016/j.econedurev.2015.03.006

[7] Su Y, Lau C, Rao N. Early education policy in China: Reducing regional and socioeconomic disparities in preschool attendance. Early Child Res Q. 2020;53: 11–22. doi: 10.1016/j.ecresq.2020.02.001

[8] Ministry of Education of the People’s Republic of China. The three-year gross enrollment rate for preschool reaches 88.1 percent, and preschool education has been basically universal [in Chinese]. 2022 [cited 2023 Jan 22]. Available from: http://www.gov.cn/xinwen/2022-04/28/content_5687602.htm#:~.

[9] Gong X, Xu D, Han W-J. The effects of preschool attendance on adolescent outcomes in rural China. Early Child Res Q. 2016;37: 140–152. doi: 10.1016/j.ecresq.2016.06.003

[10] Bietenbeck J, Ericsson S, Wamalwa FM. Preschool attendance, schooling, and cognitive skills in East Africa. Econ Educ Rev. 2019;73: 101909. doi: 10.1016/j.econedurev.2019.101909

[11] Lassassi M. Does preschool improve child development and affect the quality of parent-child interaction? Evidence from Algeria. Int J Educ Dev. 2021;82: 102354. doi: 10.1016/j.ijedudev.2021.102354

[12] Rao N, Zhou J, Sun J, editors. Early Childhood Education in Chinese Societies. Dordrecht: Springer Netherlands; 2017. doi: 10.1007/978-94-024-1004-4

[13] Bronfenbrenner U. Contexts of child rearing: Problems and prospects. Am Psychol. 1979;34: 844–850. doi: 10.1037/0003-066X.34.10.844

[14] Walk LM, Evers WF, Quante S, Hille K. Evaluation of a teacher training program to enhance executive functions in preschool children. PLOS ONE. 2018;13: e0197454. doi: 10.1371/journal.pone.0197454 29795603 PMC5967750

[15] Chen C, Ahlqvist VH, Henriksson P, Magnusson C, Berglind D. Preschool environment and preschool teacher’s physical activity and their association with children’s activity levels at preschool. PLOS ONE. 2020;15: e0239838. doi: 10.1371/journal.pone.0239838 33057340 PMC7561096

[16] Rao N, Richards B, Sun J, Weber A, Sincovich A. Early childhood education and child development in four countries in East Asia and the Pacific. Early Child Res Q. 2019;47: 169–181. doi: 10.1016/j.ecresq.2018.08.011

[17] Hu BY, Fan X, Wu Z, LoCasale-Crouch J, Yang N, Zhang J. Teacher-child interactions and children’s cognitive and social skills in Chinese preschool classrooms. Child Youth Serv Rev. 2017;79: 78–86. doi: 10.1016/j.childyouth.2017.05.028

[18] Hu BY, Roberts SK. A qualitative study of the current transformation to rural village early childhood in China: Retrospect and prospect. Int J Educ Dev. 2013;33: 316–324. doi: 10.1016/j.ijedudev.2012.04.006

[19] Coley RL, Votruba-Drzal E, Collins M, Cook KD. Comparing public, private, and informal preschool programs in a national sample of low-income children. Early Child Res Q. 2016;36: 91–105. doi: 10.1016/j.ecresq.2015.11.002

[20] Loeb S, Bridges M, Bassok D, Fuller B, Rumberger RW. How much is too much? The influence of preschool centers on children’s social and cognitive development. Econ Educ Rev. 2007;26: 52–66. doi: 10.1016/j.econedurev.2005.11.005

[21] Deming D. Early Childhood Intervention and Life-Cycle Skill Development: Evidence from Head Start. Am Econ J Appl Econ. 2009;1: 111–134. doi: 10.1257/app.1.3.111

[22] Amadon S, Gormley WT, Claessens A, Magnuson K, Hummel-Price D, Romm K. Does early childhood education help to improve high school outcomes? Results from Tulsa. Child Dev. 2022;n/a. doi: 10.1111/cdev.13752 35302656

[23] Datta Gupta N, Simonsen M. Academic performance and type of early childhood care. Econ Educ Rev. 2016;53: 217–229. doi: 10.1016/j.econedurev.2016.03.013

[24] Rao N, Sun J, Zhou J, Zhang L. Early achievement in rural China: The role of preschool experience. Early Child Res Q. 2012;27: 66–76. doi: 10.1016/j.ecresq.2011.07.001

[25] Bastos P, Straume OR. Preschool Education in Brazil: Does Public Supply Crowd Out Private Enrollment? World Dev. 2016;78: 496–510. doi: 10.1016/j.worlddev.2015.10.009

[26] Berlinski S, Galiani S, Gertler P. The effect of pre-primary education on primary school performance. J Public Econ. 2009;93: 219–234. doi: 10.1016/j.jpubeco.2008.09.002

[27] Chen S, Zhao C, Cao Y, Chen C, Snow CE, Lu M. Long-term effects of China’s One Village One Preschool program on elementary academic achievement. Early Child Res Q. 2019;49: 218–228. doi: 10.1016/j.ecresq.2019.06.010

[28] Zhang S. Effects of attending preschool on adolescents’ outcomes: evidence from China. Appl Econ. 2017;49: 2618–2629. doi: 10.1080/00036846.2016.1243217

[29] Arapa B, Sánchez E, Hurtado-Mazeyra A, Sánchez A. The relationship between access to pre-school education and the development of social-emotional competencies: Longitudinal evidence from Peru. Int J Educ Dev. 2021;87: 102482. doi: 10.1016/j.ijedudev.2021.102482

[30] Zheng L, Weng Q, Gong X. The cognitive ability Gap between preschool education and urban and rural middle school students: A study based on CEPS data [in Chinese]. Sociological research, 2019; (03): 122–145+244.

[31] Wong HL, Luo R, Zhang L, Rozelle S. The impact of vouchers on preschool attendance and elementary school readiness: A randomized controlled trial in rural China. Econ Educ Rev. 2013;35: 53–65. doi: 10.1016/j.econedurev.2013.03.004

[32] Feng S, Han Y, Heckman JJ, Kautz T. Comparing the reliability and predictive power of child, teacher, and guardian reports of noncognitive skills. Proc Natl Acad Sci—PNAS. 2022;119. doi: 10.1073/pnas.2113992119 35131849 PMC8833216

[33] Chen Y, Feng S, Heckman JJ, Kautz T. Sensitivity of self-reported noncognitive skills to survey administration conditions. Proc Natl Acad Sci U S A. 2020;117: 931–935. doi: 10.1073/pnas.1910731117 31888989 PMC6969507

[34] Gong X, Xu D, Han W-J. Household Income and Preschool Attendance in China. Child Dev. 2015;86: 194–208. doi: 10.1111/cdev.12294 25174375

[35] Chinese Research Association of Preschool Education. Compilation of important literature on early childhood education in the People’s Republic of China [in Chinese]. Beijing: Beijing Normal University Press; 1999.

[36] Tian J, Zhou D, Zhang S. 70 years of preschool education in the People’s Republic of China [in Chinese]. Changsha: Hunan University Press; 2020.

[37] Tang S. The development and reform of early childhood education in Rural China [in Chinese]. Studies in Early Childhood Education, 2005; 06: 38–40.

[38] Wu KB, Young ME, Cai J. Early Child Development in China: Breaking the Cycle of Poverty and Improving Future Competitiveness. Washington, DC: The World Bank; 2012. Available: https://go.exlibris.link/4xcBV99q.

[39] Ministry of Education of the People’s Republic of China (MOE). Educational statistics yearbook of China in 2019 [in Chinese]. Beijing: People’s Education Press; 2020.

[40] The Development and Reform commission of Qinghai province, the 13th Five-Year Plan of Education development [in Chinese]. 2016 [cited 2023 Jan 22]. Available from: http://fgw.qinghai.gov.cn/ztzl/n2018/sswgh/sswzxgh/201602/t20160219_50995.html.

[41] Ministry of education of Ningxia Hui Autonomous Region, The 13th Five-Year Plan for the Development of Education in Ningxia Hui Autonomous Region [in Chinese]. 2021 [cited 2023 Jan 22]. Available from: http://jyt.nx.gov.cn/zwgk/zfxxgkml/ghjh/202201/t20220106_3274908.html.

[42] National Bureau of Statistics of the People’s Republic of China. China statistics yearbook in 2010 [in Chinese]. Beijing: China Statistics Press; 2011.

[43] National Rural Revitalization Administration, National progress in poverty alleviation [in Chinese]. 2014 [cited 2023 Jan 22]. Available from http://nrra.gov.cn/art/2014/12/23/art_343_981.html.

[44] Zhou B. Mental health test [in Chinese]. Shanghai: East China Normal University Press; 1991.

[45] Nunnelly J. C., Bernstein I. Psychometric theory. New York: McGraw Hill; 1978.

[46] Imbens GW, Rubin DB. Causal Inference in Statistics, Social, and Biomedical Sciences. Cambridge: Cambridge University Press; 2015.

[47] Oster E. Unobservable Selection and Coefficient Stability: Theory and Evidence. J Bus Econ Stat. 2019;37: 187–204. doi: 10.1080/07350015.2016.1227711

[48] Heckman J, Pinto R, Savelyev P. Understanding the Mechanisms Through Which an Influential Early Childhood Program Boosted Adult Outcomes. Am Econ Rev. 2013;103: 2052–2086. doi: 10.1257/aer.103.6.2052 24634518 PMC3951747

[49] Gupta ND, Simonsen M. Non-cognitive child outcomes and universal high quality child care. J Public Econ. 2010;94: 30–43. doi: 10.1016/j.jpubeco.2009.10.001

[50] Blanden J, Del Bono E, McNally S, Rabe B. Universal pre-school education: The case of public funding with private provision. Econ J Lond. 2016;126: 682–723. doi: 10.1111/ecoj.12374

[51] Barnett WS. Benefits of Compensatory Preschool Education. J Hum Resour. 1992;27: 279. doi: 10.2307/145736

[52] Luo R, Zhang L, Liu C, Zhao Q, Shi Y, Rozelle S, et al. Behind before they Begin: The Challenge of Early Childhood Education in Rural China. Australas J Early Child. 2012;37: 55–64. doi: 10.1177/183693911203700107

[53] Okitsu T, Edwards DB, Mwanza P, Miller S. Low-fee private preschools as the symbol of imagined ‘modernity’?–Parental perspectives on early childhood care and education (ECCE) in an urban informal settlement in Zambia. Int J Educ Dev. 2023;97: 102723. doi: 10.1016/j.ijedudev.2022.102723

[54] Wang L, Dang R, Bai Y, Zhang S, Liu B, Zheng L, et al. Teacher qualifications and development outcomes of preschool children in rural China. Early Child Res Q. 2020;53: 355–369. doi: 10.1016/j.ecresq.2020.05.015

[55] Su Y, Rao N, Sun J, Zhang L. Preschool quality and child development in China. Early Child Res Q. 2021;56: 15–26. doi: 10.1016/j.ecresq.2021.02.003

